# Supplementary material for: Accurate informatic modeling of tooth enamel pellicle interactions by training substitution matrices with Mat4Pep
Source: Front Mater. Author manuscript; Available in PMC 2025 Aug 2. (PMC12317685; doi:10.3389/fmats.2024.1436379)
Supplement: Supplementary material [file NIHMS2095415-supplement-Supplementary_material.doc]

| Sequence Analysis  **Informatic modeling of tooth enamel pellicle interactions**  Jeremy Horst Keeper^1,2^, Jong Seto^2,3,4*^, Ersin Emre Oren^5^, Orapin V. Horst^6^, Ling-Hong Hung^7^ and Ram Samudrala^8^  ^1^Oral Health Sciences, University of Washington, WA, USA; ^2^Department of Biochemistry and Biophysics, University of California, San Francisco, CA, USA; ^3^School for Engineering of Matter, Transport, and Energy Center of Biological Physics, Arizona State University, Tempe, AZ USA; ^4^Molecular Foundry, Lawrence Berkeley National Laboratory, Berkeley, CA USA; ^5^Bionanodesign Laboratory, Department of Biomedical Engineering, TOBB University of Economics and Technology, Ankara, Turkey; ^6^Department of Preventive and Restorative Dental Sciences, University of California, San Francisco, CA, USA; ^7^Institute of Technology, University of Washington, Tacoma, WA, USA; ^8^Department of Biomedical Informatics, State University of New York, Buffalo, NY, USA. |
| --- |

SupPlementary materials

### Contents

**Figure S1 |** Weak selection by commonly used protein sequence comparison algorithms.

**Figure S2 |** Relationship of pre- & post-training accuracy to matrix distance.

**Figure S3 |** Relationship of improvement in accuracy to magnitude of change in matrices.

**Figure S4 |** Relationship of post-training accuracy to pre-training accuracy.

**Table S1 |** Nonredundant pellicle peptide sequences.

**Table S2 |** Control set.

**Table S3 |** Additional control set derived from random parts of human proteins.

**Table S4 |** Additional control set derived from combinations of residues randomized to the incidence in UniProt.

**Table S5 |** The β-3D-Ali trained matrix.

**Table S6 |** Selection of the β-3D-Ali matrix.

**Table S7 |** Protein binding region recapture by matrix.

**Table S8 |** Analysis of the β-3D-Ali trained matrix.

**Table S9 |** Novel pellicle peptides.

# SUPLEMENTARY FIGURES

**Figure S1 | Weak selection by commonly used protein sequence comparison algorithms.** PSI-BLAST (dar blue) shows weak discriminatory power, while HHpred (yellow) and Neural Networks (orange; SKLearn) show no ability to separate enamel pellicle peptides from controls in a modified leave-one-out experiment. a. Receiver operating characteristic curve. b. Precision recall curve. c. Matthews correlation coefficient (MCC) curve. d. scores of pellicle peptides (red) and controls (blue). This result indicates that there are no strongly conserved position-specific patterns in the pellicle peptides to enable construction of the multiple sequence alignments required by commonly used protein sequence comparison algorithms, and reveals the subtlety of the patterns recognized by the substitution matrices.

#

# Figure S2 | Relationship of pre- & post-training accuracy to matrix distance. All matrices were run through the training protocol described in Results section 3.4 Refinement. Pre-training accuracies by the area under the receiver operating characteristic curve are shown in blue and post-training accuracies are shown in black, connected by a blue line for each matrix. These are plotted against the arithmetic distance for each matrix pre- and post-training. While some matrices can be trained with the protocol to reach the global maximum, or near to it, others get stuck in local maxima. There is a 0.44 (Pearson’s R) correlation between post-training accuracy and change (distance) in the matrix from training.

#

# Figure S3 | Relationship of improvement in accuracy to magnitude of change in matrices. All matrices were run through the training protocol described in Results section 3.4 Refinement. The correlation of the arithmetic distance to improvement in accuracy is 0.55 (Pearson’s R).

#

# Figure S4 | Relationship of post-training accuracy to pre-training accuracy. All matrices were run through the training protocol described in Results section 3.4 Refinement.

#

# SUPLEMENTARY TABLES

**Table S1 | Nonredundant pellicle peptide sequences.** The 78 peptides identified by Siqueira and Oppenheim were aligned by hand, and filtered for redundancy. The resulting set includes 49 peptides, eight to 36 residues in length.

P01036_1: EVPWEDRMSLVN

P01040_1: KTNETYGKLE

P01040_2: TYGKLEAVQY

P01040_3: SLPGQNEDLVLTG

P01833_1: ASVDSGSSEEQGGSSRAL

P02647_1: REQLGPVTQEF

P02768_1: PLVEEPQNLIK

P02768_2: AVMDDFAAFVEK

P02788_1: ESTVFEDLSDEAER

P02808_2: DSSEEKFLR

P02808_5: RIGRFGYGYGPYQPVPEQPLYPQPYQPQYQQYT

P02810_1: IDEERQGPPLGGQQSQPS

P02810_11: GPPPPPPGKPQGPPPQGGRPQGPPQGQSPQ

P02810_13: GPPQQGGHQQGPPPPPPGKPQ

P02810_2: DDGPQQGPPQQGGQQQQGPPPPQGKPQ

P02812_2: GKPQGPPPQGGNQPQGPPPPPGKPQGPPPQGGNKPQ

P04080_1: QTNKAKHDELTYF

P04083_1: FIENEEQEYVQTVK

P04083_2: TPAQFDADELR

P04280_2: PQGPPPQGGNKPQGPPPPGKPQGPPPQGDK

P04406_1: SEGPLKGILGY

P04406_2: SSDFNSDTHSSTF

P04745_1: SGNEDEFR

P04745_2: ALVFVDNHDNQR

P04745_3: IYQEVIDLGGEPIK

P04745_4: AHFSISNSAEDPFIAI

P04745_5: TGSGDIENYNDATQVR

P05164_1: DFVNCSTLPALNLASW

P06702_1: SVKLGHPDTLNQGEFKEL

P06733_1: NGWGVMVSH

P06733_2: SERLAKYNQLLRIEEE

P08779_1: EVFTSSSSSSSRQ

P15515_1: YGDYGSNYLYDN

P20671_1: RNDEELNKLLGKVTIA

P30044_1: APIKVGDAIPAVEV

P31151_1: HKQSHGAAPCSGGS

P47929_1: GGDVQLDSVRIF

P62736_1: VGDEAQSKRGILTL

P62736_2: AASSSSLEKSYELPDGQVI

Q01546_1: GVFGGVSGSGSGGYK

Q01546_2: SGGGGSTSIRFSQTTSSSQHSSTK

Q96QV6_1: TIAQGGVLPNIQAV

Q9HC84_1: SSPSPAPGCDNAIP

Q9HC84_2: AQAQPGVP

Q9HCY8_1: NFHQYSVEGG

Q9HCY8_2: RSFWELIGEAAKSVKLE

Q9HCY8_4: QEFSDVERAIETLI

Q9HCY8_5: IANLGSCNDSKL

Q9UBG3_1: TEGNCTALTRGELKR

**Table S2 | Control set.** Peptide sequences were derived from the same 29 proteins that are the source of the pellicle peptides used in the experiments. Random starting points were attempted throughout each protein sequence, iteratively searching for areas not overlapping with any of the 78 pellicle peptide regions, and of equivalent length and quantity to the pellicle peptides from the same protein. If it were not possible to derive control sequences of equal length and quantity in the same protein, because of coverage by the pellicle peptide regions, a random alternate source protein was used.

P01036_1 ATLAGALASSSK

P01040_1 IVDKVKPQLE

P01040_2 SEAKPATPEI

P01040_3 TPEIQEIVDKVKP

P01833_1 GSFSVVITGLRKEDAGRY

P02647_1 SQFEGSALGKQ

P02768_1 PNLPRLVRPEV

P02768_2 CTVATLRETYGE

P02788_1 QGLKSCHTGLRRTA

P02808_1 EEKFLRRIG

P02808_2 MKFLVFAFILALMVSMIGADSSEEKFLRRIGRF

P02810_1 LLILLSVALLAFSSAQDL

P02810_2 LLSVALLAFSSAQDLDEDVSQEDVPLVISD

P02810_3 AFSSAQDLDEDVSQEDVPLVI

P02810_4 SSAQDLDEDVSQEDVPLVISDGGDSEQ

P04080_1 SQLEEKENKKFPV

P04083_1 LRRVFQKYTKYSKH

P04083_2 AMVSEFLKQAW

P04280_1 LLILLSVALLALSSAQNLNEDVSQE

P04280_2 LILLSVALLALSSAQNLNEDVSQEESPSLI

P04406_1 DPSKIKWGDAG

P04406_2 FQYDSTHGKFHGT

P04745_1 FWLLFTIG

P04745_2 ALECERYLAPKG

P04745_3 IHNPFRPWWERYQP

P04745_4 RTSIVHLFEWRWVDIA

P04745_5 MKLFWLLFTIGFCWAQ

P05164_1 TSFVDASMVYGSEEPL

P06702_1 MTCKMSQLERNIETIINT

P06733_1 QLADLYKSF

P06733_2 FRSGKYDLDFKSPDDP

P08779_1 GIGGGSSRISSVL

P15515_1 FFVFALVLALMI

P20671_1 RDNKKTRIIPRHLQLA

P30044_1 YILVGGAGGQSAAA

P31151_1 MIDMFHKYTRRDDK

P47929_1 SWGREERGPGVP

P62736_2 CEEEDSTALVCDNGSGLCK

Q01546_1 VCISVVSNVTSTSGS

Q01546_2 RSGGAGGGACGFRSGAGSFGSRSL

Q96QV6_1 AAVLEYLTAEILEL

Q9HC84_1 NWEQEGVFKMCYNY

Q9HC84_2 HTPPVPNT

Q9HCY8_1 AEDAQEFSDV

Q9HCY8_2 AEDAQEFSDVERAIETL

Q9HCY8_3 ANAEDAQEFSDVER

Q9HCY8_4 QCRSANAEDAQE

Q9UBG3_1 LQNINGIIEAFRRYA

**Table S3 | Additional control set derived from random parts of human proteins.** Peptide sequences were derived randomly from the human proteome to match the size and quantity of the pellicle peptide set used in the experiments.

1 PKEPPSLDQLRA

2 NWDLQQGLLR

3 CGLNKLEKRD

4 TVLLEVKFIQGNL

5 KAEEKAKEIAKMAEMLVE

6 PPGNPQSIYYH

7 YLKSLKQEFEM

8 VKRPSPESQSNN

9 LPIWGHFPAAARTR

10 VPDRMCRHN

11 GSREDSPMSPSDTQDQKRTLRPPSRHGHSVVAP

12 PPSGNQNGAEGDQINASK

13 KSQEGQGSILETITLIQDCKDTLELIETEL

14 SADELQKMLQEDELRDAVLLV

15 ETIKLGNLPTGSFSSSSPSSSSSRQTV

16 SVDSPALTRFFTFHFILPFIIAALAALHLLFLHETG

17 AQPLPPPHGTASL

18 QQQGDTPAVSSSVT

19 YKSFCREEKEW

20 PPGHQDVSERRLDTQRPQVTMWERD

21 RNPSRSTCPTWWYTMQAPTSSRGTALGGCP

22 AGMNAAVRSAV

23 SFDLGRQFLFHWT

24 SRRDDLES

25 QEGIQALPYQLS

26 YSPQMQSAPKRWRE

27 LFLFTTPCRLVRPVQN

28 ANISVLFGFINFFLWA

29 TGAWIFTGGVNTGVIR

30 TLLVNRKDAMWVPCLVSI

31 QAATPKIFN

32 YVDDTLFVRFDSDATS

33 AAFSPTFPIYMVF

34 NGVQLQQQKNKEMEQL

35 PQGLPGSRGELGPV

36 LSCLLGPPEAASQG

37 ETRPAGDRTFQK

38 SIQSLLDEPNPNSP

39 YDSDWNPQVDLQAMDRAHR

40 HEVNELTSSRLLKLE

41 KHWSGFLREAFTDADNFGIQFPRD

42 DNIPCKKCVVVGNG

43 QVIQSLIALVNDPQ

44 DFQEERDF

45 KIPVRSQKRT

46 SRLDSCIWKLLVKALYS

47 VSPTVLDMWGQEGT

48 PPLDPTPFPNSF

49 SLSIEDTFESISELG

**Table S4 | Additional control set derived from combinations of residues randomized to the incidence in UniProt.** Contrived peptide sequences were built from sequential combination of amino acids randomly selected to match the probability distribution of each residue type in UniProt to match the size and quantity of the pellicle peptide set used in the experiments.

1 NETMPLRARYGH

2 AANPLIVMCM

3 FRATRAWMEL

4 ILYTIFPKVCPPE

5 RNVGLCVTLNDNQELALK

6 GKADTLSHGSV

7 FGRLTLSRGLS

8 IPDLRDDRDRVI

9 LELSTAALPLLGTM

10 ARILVGHES

11 CQDAGDIPQEYCPVGEPAEGNRKSDADCTGCLG

12 ETASEPESFDLWFGVADN

13 ILVAMGTNFLTEYDSTRESQCTPYKTTRFV

14 WNLQCPNATFKTQRQVGDSNE

15 LAEYLSCPRTLESFRFYMADDLPGYRA

16 DREPAAKSKQGGLRETDSKSSLAWDQVQGQLFKVRT

17 LVTAVYPGPMINS

18 PSPCTEFLGANANA

19 TLGLRKELKPP

20 ANSLYGPNIEAKKQILMPEPAYAGS

21 KKASQLHMVDRTNVKLHSKITGQMLRVDGR

22 SIIDVLRDHGH

23 VWTRVDAGSLGDF

24 NTSQSTPA

25 MMVFPQLLRRHD

26 IILPEEASSAGPKA

27 RLPHGRMPVEQPEMLC

28 AVKEGVVNTAPPLHQD

29 WKPDGPPDKRHKITLK

30 ASGGRGNAPPSSWGGILA

31 NYLRRPKDM

32 MTMEKPDGSRSEYVFW

33 TTQDPIDDLGTRL

34 HPHETLLDRLVFAETF

35 GWRETPQTYLALEY

36 QDVVAIPIMFQIDE

37 YVNDTYMSLDLP

38 VHLIIEEEKNAPKE

39 MAISTGSSPKAAAQALHAQ

40 PDTGREPGESTILDG

41 AFYDYRYAMGKVHIRQETDPPQYQ

42 GLQDARLHGHGYDG

43 EKDLQGPDGELTND

44 DSISSDKR

45 STSFVFGSAE

46 QMLVIAAERTSPYGHTY

47 CADLEDPVSLVYRP

48 VPYLMIYELQDP

49 TGRQYALEAHKQPYK

**Table S5 | The β-3D-Ali trained matrix. A.** Shown are the matrix values used for pairwise comparison of query to target (pellicle peptide / control sequence) amino acids. Selection of the β-3D-Ali matrix indicates a strong preference of the pellicle peptides for β-strand-like structures. **B.** The differences from the original values after training the matrix to more accurately separate enamel pellicle peptides from controls.

0.98  **A** 0.75

0.72  **R** 0.80 -2.00

1.09  **N** 0.71 2.00 0.80

0.77  **D** **0.86** 2.00 0.69 0.66

0.57  **C** 0.75 0.84 0.72 -2.00 1.07

0.72  **Q** 0.68 0.83 0.71 0.64 0.65 0.80

0.93  **E** 0.63 0.82 0.74 0.58 0.69 0.73 2.00

0.96  **G** **1.63** 2.00 0.71 0.61 0.80 0.78 0.87 0.97

0.81  **H** 0.79 0.92 0.78 0.68 -2.00 0.79 0.78 0.71 0.99

1.16  **I** 2.00 1.23 1.12 2.00 0.96 1.10 -2.00 2.00 1.24 1.33

1.02  **L** 0.97 1.05 0.97 2.00 0.84 1.07 1.12 2.00 1.07 1.21 **-1.40**

0.71  **K** 0.65 -2.00 2.00 0.64 0.79 0.75 0.78 0.76 0.82 1.11 **1.69** 0.78

0.60  **M** 2.00 1.14 0.92 0.93 0.97 -2.00 2.00 1.18 0.96 2.00 1.06 -2.00 1.05

1.07  **F** 1.16 **-0.60** 2.00 1.17 1.04 1.07 1.17 1.30 1.05 1.54 1.27 1.11 -2.00 1.33

0.63  **P** 0.77 1.03 2.00 0.75 0.67 -2.00 2.00 -2.00 0.83 1.30 1.21 0.92 **-0.80** 2.00 0.82

1.02  **S** -2.00 0.99 0.84 0.77 **1.51** 0.85 0.92 **0.51** 0.85 1.24 2.00 0.89 1.15 1.28 **1.17** 0.96

1.09  **T** 2.00 2.00 0.99 2.00 0.93 1.02 1.11 1.03 2.00 **-0.80** 1.18 1.09 2.00 1.32 0.93 1.10 1.16

0.79  **W** 1.18 1.33 0.95 -2.00 **-1.10** 2.00 1.33 1.28 1.18 1.71 1.37 1.11 **-1.10** 1.31 1.09 2.00 **-1.90** 1.22

0.96  **Y** 1.23 -2.00 1.08 **1.42** 1.22 2.00 1.31 1.27 **0.63** 1.51 1.41 1.18 1.23 1.34 -2.00 1.41 1.46 1.29 1.31

1.34  **V** 2.00 2.00 1.05 0.99 2.00 2.00 1.08 1.09 **1.17** 1.42 **-1.70** 1.06 1.22 1.51 2.00 2.00 1.27 1.63 1.60 1.41

**A R N D C Q E G H I L K M F P S T W Y V**

**mean** 0.98 0.72 1.09 0.77 0.57 0.72 0.93 0.98 0.81 1.16 1.02 0.71 0.60 1.07 0.63 1.02 1.09 0.79 0.96 1.34

**Supplemental Table 5a.**

**A** 0.0

**R** 0.0 2.9

**N** 0.0 -1.12 0.0

**D** **-0.3** -1.37 0.0 0.0

**C** 0.0 0.0 0.0 2.67 0.0

**Q** 0.0 0.0 0.0 0.0 0.0 0.0

**E** 0.0 0.0 0.0 0.0 0.0 0.0 -1.26

**G** **-0.8** -1.16 0.0 0.0 0.0 0.0 0.0 0.0

**H** 0.0 0.0 0.0 0.0 2.69 0.0 0.0 0.0 0.0

**I** -0.89 0.0 0.0 -0.94 0.0 0.0 3.15 -0.75 0.0 0.0

**L** 0.0 0.0 0.0 -1.04 0.0 0.0 0.0 -0.91 0.0 0.0 **2.52**

**K** 0.0 2.84 -1.28 0.0 0.0 0.0 0.0 0.0 0.0 0.0 **-0.7** 0.0

**M** -1.03 0.0 0.0 0.0 0.0 2.97 -1.04 0.0 0.0 -0.84 0.0 3.03 0.0

**F** 0.0 **1.86** -0.89 0.0 0.0 0.0 0.0 0.0 0.0 0.0 0.0 0.0 3.21 0.0

**P** 0.0 0.0 -1.09 0.0 0.0 2.83 -1.25 2.75 0.0 0.0 0.0 0.0 **2.14** -0.93 0.0

**S** 2.8 0.0 0.0 0.0 **-0.7** 0.0 0.0 **0.4** 0.0 0.0 -0.91 0.0 0.0 0.0 **-0.3** 0.0

**T** -1.03 -0.92 0.0 -1.12 0.0 0.0 0.0 0.0 -1.09 **2.07** 0.0 0.0 -0.89 0.0 0.0 0.0 0.0

**W** 0.0 0.0 0.0 3.04 **2.57** -0.83 0.0 0.0 0.0 0.0 0.0 0.0 **2.45** 0.0 0.0 -0.66 **3.32** 0.0

**Y** 0.0 3.25 0.0 **-0.3** 0.0 -0.89 0.0 0.0 **0.6** 0.0 0.0 0.0 0.0 0.0 3.29 0.0 0.0 0.0 0.0

**V** -0.95 -0.92 0.0 0.0 -1.09 -0.97 0.0 0.0 **-0.04** 0.0 **3.05** 0.0 0.0 0.0 -0.87 -0.79 0.0 0.0 0.0 0.0

**A R N D C Q E G H I L K M F P S T W Y V**

**Supplemental Table 5b.Table S6 | Selection of the β-3D-Ali matrix.** Selection of the β-3D-Ali matrix indicates a strong preference of the pellicle peptides for β-strand-like structures.

AAindex name gop gep ROC AUC Student's T-test

Amino acid distribution 0.571455 0.010214850

1. MEHP950102 -5 -2 0.924941 3.44095*10^-15

2. FITW660101 -8 -7 0.898479 5.15462*10^-15

3. MEHP950103 -7 -1 0.890563 2.99985*10^-13

4. WEIL970102 -4 -3 0.870570 4.82138*10^-12

5. MIYT790101 -16 -6 0.869413 1.69328*10^-12

6. MUET010101 -9 -3 0.834132 4.10195*10^-09

7. ALTS910101 -5 -1 0.833628 7.07831*10^-09

8. DAYM780302 -5 -1 0.823590 1.19290*10^-07

9. CROG050101 -9 -1 0.822309 2.12069*10^-08

10. OVEJ920101 -4 -3 0.815419 2.31327*10^-08

11. JOHM930101 -8 -3 0.812983 4.80010*10^-08

12. FEND850101 -16 -8 0.793368 1.44116*10^-07

13. PRLA000102 -15 -1 0.788663 5.74555*10^-07

14. HENS920101 -7 -2 0.787656 2.19269*10^-06

15. HENS920103 -2 -7 0.785997 4.72036*10^-07

16. MEHP950101 -3 -3 0.779465 1.06235*10^-06

17. HENS920104 -10 -1 0.777218 9.12226*10^-07

18. HENS920102 -7 -4 0.765184 6.71431*10^-07

19. DAYM780301 -4 -4 0.762180 4.99556*10^-07

20. GRAR740104 -6 -1 0.760899 2.98544*10^-06

21. MUET020101 -9 -1 0.756888 9.68398*10^-07

22. PRLA000101 -3 -1 0.755545 5.13833*10^-06

23. WEIL970101 -3 -3 0.751136 1.51861*10^-06

24. BENS940103 -5 -2 0.749056 6.19061*10^-06

25. DOSZ010104 -6 -4 0.747521 1.10033*10^-05

26. HORJ100101 -16 -1 0.740382 5.27549*10^-06

27. MCLA710101 -16 -8 0.736056 3.89421*10^-05

28. NGPC000101 -7 -2 0.733389 9.70233*10^-06

29. BENS940102 -1 -5 0.731268 1.42807*10^-05

30. RIER950101 -16 -8 0.730239 2.12551*10^-05

31. GONG920101 -4 -3 0.728264 1.17571*10^-05

32. MUET020102 -8 -2 0.723456 1.29363*10^-05

33. BLAJ010101 -15 -7 0.721732 4.36085*10^-05

34. GEOD900101 -14 -7 0.719148 3.40237*10^-05

35. KAPO950101 -16 -8 0.717952 0.000156902

36. BENS940101 -9 -1 0.697097 4.25286*10^-05

37. DOSZ010102 -3 -1 0.688361 0.000307285

38. JOND920103 -2 -6 0.680066 7.46935*10^-05

39. MOHR870101 -15 -3 0.674459 0.001385479

40. OGAK980101 -1 -1 0.670278 0.003566957

41. LEVJ860101 -1 -2 0.666729 0.000108593

42. VOGG950101 -14 -7 0.660557 0.002832480

43. OVEJ920104 -2 -1 0.657660 0.220723040

44. LINK010101 -2 -1 0.657660 0.220723040

45. RISJ880101 -6 -5 0.657614 0.005727649

46. OVEJ920105 -2 -1 0.653333 0.116641516

47. BENS940104 -4 -4 0.643208 0.000882362

48. RUSR970101 -12 -8 0.637517 0.017027975

49. RUSR970102 -4 -5 0.632350 0.004573817

50. QUIB020101 -12 -1 0.629768 0.002164891

51. QU_C930103 -10 -4 0.622017 0.036218923

52. KANM000101 -16 -8 0.612820 0.001669833

53. NAOD960101 -15 -8 0.599462 0.010331570

54. JOND940101 -11 -7 0.595766 0.006558219

55. DOSZ010103 -2 -8 0.576423 0.047392014

56. AZAE970102 -15 -8 0.566490 0.021444273

57. GIAG010101 -3 -5 0.556809 0.181691107

58. DOSZ010101 -16 -8 0.555486 0.167190766

59. KOSJ950103 -16 -2 0.550341 0.428519836

60. NIEK910101 -2 -1 0.548556 0.085682058

61. CSEM940101 -2 -1 0.548094 0.064262364

62. MCLA720101 -11 -4 0.545972 0.379957446

63. KOLA920101 -16 -8 0.545469 0.201715605

64. KOSJ950111 -12 -4 0.544208 0.464242377

65. QU_C930102 -1 -2 0.543327 0.158554015

66. KOSJ950114 -16 -7 0.542403 0.413211095

67. KOSJ950115 -16 -6 0.542065 0.424915138

68. NIEK910102 -2 -1 0.542003 0.105855144

69. TUDE900101 -14 -8 0.541707 0.470630866

70. AZAE970101 -16 -8 0.538160 0.049520978

71. MIYS930101 -3 -1 0.535074 0.408049971

72. KOSJ950107 -14 -7 0.531187 0.418971016

73. QU_C930101 -2 -1 0.529046 0.129084040

74. RUSR970103 -6 -8 0.522535 0.287346652

75. KOSJ950113 -16 -8 0.504052 0.256583719

**Table S7 | Protein binding region recapture by matrix.** Accuracy values are shown for relative amino acid content, before and after training for the β-3D-Ali and PAM250 matrices, and the matrices which performed in the top 10 for pellicle peptide discrimination.

**Matrix AUC**

Amino acid 0.62296

β-3D-Ali.trained 0.747826

β-3D-Ali 0.729073

FITW660101 0.388413

MEHP950103 0.395533

WEIL970102 0.447691

MIYT790101 0.674203

MUET010101 0.656208

ALTS910101 0.265071

DAYM780302 0.393519

CROG050101 0.296515

OVEJ920101 0.49497

JOHM930101 0.406083

PAM250.trained 0.599935

PAM250 0.30579

**Table S8 | Analysis of the β-3D-Ali trained matrix.** A. Difference between the pairwise amino acid score for the identical residue and the average (mean, μ) of all possible residue substitutes. These data indicate the preference for matching the particular amino acid, where glutamic acid is highly important to be matched to a glutamic acid across enamel pellicle proteins but self match is penalized for leucine and arginine. B. The amino acid pair values altered in training the β-3D-Ali matrix to more accurately separate enamel pellicle peptides from controls. The differences from the original values after training the matrix suggest unique features of these enamel binding peptides relative to other β-strand-like structures.

**Self-μ Difference Final**

**E** 1.07 **GA** -0.80 1.63

**C** 0.50 **KL** -0.70 1.69

**M** 0.45 **SC** -0.70 1.51

**W** 0.43 **YD** -0.30 1.42

**Y** 0.35 **SP** -0.30 1.17

**F** 0.26 **DA** -0.30 0.86

**P** 0.19 **VH** -0.04 1.17

**H** 0.18 **SG** 0.40 0.51

**I** 0.17 **YH** 0.60 0.63

**Q** 0.08 **FR** 1.86 -0.60

**T** 0.07 **TI** 2.07 -0.80

**K** 0.07 **PM** 2.14 -0.80

**V** 0.07 **WM** 2.45 -1.10

**G** 0.01 **LL** 2.52 -1.40

**S** -0.06 **WC** 2.57 -1.10

**D** -0.11 **VL** 3.05 -1.70

**A** -0.23 **WT** 3.32 -1.90

**N** -0.29

**L** -2.42

**R** -2.72

**Table S8a. Table S8b.**

**Table S9 | Novel pellicle peptides.** Peptides were identified by iST mass spectrometry from pooled enamel pellicle samples. Peptides with pellitrix scores over that of the highest scoring control sequence are shown.

| **Peptide sequence** | **Source protein** | **Pellitrix** | **Z-score** | **MS/MScount** | **Proteus** |
| --- | --- | --- | --- | --- | --- |
| TDLEMQIEGLK | KRT16;KRT19 | 88.6 | 5.7 | 2 | 2 |
| VFDKDGNGFVSAAELR | CALML3 | 77.8 | 4 | 3 | 3 |
| VFQSLPHENKPLTLSNYQTNKAK | CSTB | 77 | 3.8 | 4 | 4 |
| ILTATIENNR | KRT13 | 75.7 | 3.6 | 8 | 2 |
| TPGAVNACHLSCSALLQDNIADAVACAK | LYZ | 74.9 | 3.5 | 4 | 3 |
| HLEEEEGQLK | IVL | 74.1 | 3.4 | 2 | 2 |
| TTFANVVNVCGNQSIR | RNASE3 | 73.2 | 3.2 | 4 | 2 |
| AGLEDLQVAFR | CALML5 | 71.8 | 3 | 2 | 2 |
| FKVEESYDLKDTLR | SERPINB3 | 71.7 | 3 | 4 | 3;4 |
| VFSNGADLSGVTEEAPLKLSK | SERPINA1 | 71.4 | 3 | 5 | 3 |
| ACPLDQAIGLLVAIFHK | S100A6 | 71 | 2.9 | 4 | 2;3 |
| VSLDVNHFAPDELTVK | HSPB1 | 70.7 | 2.8 | 9 | 2;3 |
| ALVLIAFAQYLQQCPFEDHVK | ALB | 69.7 | 2.7 | 6 | 3;4 |
| NKLAELEEALQKAK | KRT5 | 68.7 | 2.5 | 2 | 3 |
| VAAEDWK | IGHA1;IGHA2 | 68.7 | 2.5 | 3 | 2 |
| AACLLPKLDELR | ALB | 68.6 | 2.5 | 2 | 3 |
| FASFIDKVR | KRT5;KRT8;KRT6A;KRT75;KRT6C;KRT6B;KRT84;KRT3;KRT2 | 68.2 | 2.5 | 23 | 2;3 |
| TSIVHLFEWR | AMY1A;AMY2B;AMY2A;AMY1B | 68.2 | 2.5 | 6 | 2;3 |
| FTCTVTHTDLPSPLKQTISRPK | IGHM | 68 | 2.4 | 4 | 4;5 |
| AAQGLLACGVAQGALR | PGLYRP1 | 67.8 | 2.4 | 6 | 2 |
| ASGVPDRFSGSGSGTDFTLK | IGKV2-40;IGKV2D-28 | 67.8 | 2.4 | 8 | 3 |
| VDALMDEINFMK | KRT5 | 67.7 | 2.4 | 7 | 2 |
| FSGSNSGNTATLTISR | IGLV3-9 | 67 | 2.3 | 3 | 2 |
| AEFAEVSKLVTDLTK | ALB | 66.9 | 2.3 | 7 | 2;3 |
| GGSISGGGYGSGGGKHSSGGGSR | KRT2 | 66.9 | 2.3 | 4 | 3;4 |
| MYGISLCQAILDETKGDYEK | ANXA1 | 66.8 | 2.2 | 6 | 3 |
| SQYEQLAEQNR | KRT10 | 66.6 | 2.2 | 4 | 2 |
| LGNGVQCLAMGWGLLGR | ELANE | 66.3 | 2.2 | 2 | 2 |
| ISIGGGSCAISGGYGSR | KRT6A;KRT6C;KRT6B | 66.3 | 2.2 | 20 | 2;3 |
| TDLEMQIESLNEELAYMK | KRT13 | 66.3 | 2.2 | 8 | 2;3 |
| VFLENVIRDAVTYTEHAK | HIST1H4A | 66.2 | 2.1 | 3 | 4 |
| VFAIPPSFASIFLTK | IGHM | 66.2 | 2.1 | 2 | 2 |
| LASDLLEWIRR | ACTN1;ACTN4 | 66.1 | 2.1 | 2 | 3 |
| CLDPVDTPNPTR | SLPI | 66.1 | 2.1 | 15 | 2 |
| TKKQELSEAEQATR | C3 | 65.9 | 2.1 | 3 | 2;3 |
| TLNNKFASFIDKVR | KRT5;KRT8;KRT6A;KRT75;KRT6C;KRT6B;KRT84;KRT3;KRT2 | 65.9 | 2.1 | 25 | 2;3;4 |
| ETYGEMADCCAK | ALB | 65.6 | 2 | 7 | 2 |
| AALTRDPQFQK | GPI | 65.5 | 2 | 3 | 2 |
| GGCASGLYPDAFAPVAQFVNWIDSIIQR | ELANE | 65.4 | 2 | 3 | 3 |
| KDAEAWFTSR | KRT19 | 65.3 | 2 | 2 | 3 |
| AVRPIEVTQACASPGTSCR | KLK14 | 65.1 | 2 | 3 | 3 |
| VLETKWNLLQQQTTTTSSK | KRT4 | 64.8 | 1.9 | 6 | 3 |
| SISISVAR | KRT1 | 64.7 | 1.9 | 8 | 2 |
| GGHFCGATLIAPNFVMSAAHCVANVNVR | ELANE | 64.7 | 1.9 | 3 | 4 |
| YEEEINKR | KRT4 | 64.6 | 1.9 | 9 | 2;3 |
| GGSGGSYGGGGSGGGYGGGSGSR | KRT9 | 64.5 | 1.9 | 8 | 2 |
| RENTQQHITARR | CTSG | 64.4 | 1.9 | 3 | 3;4 |
| SLVNLGGSK | KRT1 | 64.3 | 1.8 | 7 | 2 |
| TEELNKEVASNSELVQSSR | KRT16 | 64.2 | 1.8 | 15 | 2;3 |
| GGCITLISSEGYVSSK | PIGR | 64.1 | 1.8 | 3 | 2 |
| TRFVYHLSDLCK | IGJ;JCHAIN | 64.1 | 1.8 | 5 | 2;3;4 |
| FGQGSGPIVLDDVR | DMBT1 | 64 | 1.8 | 9 | 2 |
| ADYEKHKVYACEVTHQGLSSPVTK | IGKC | 63.9 | 1.8 | 20 | 3;4;5 |
| TDLEMQIEGLKEELAYLR | KRT16 | 63.9 | 1.8 | 12 | 2;3 |
| VDALIDEIDFLR | KRT3 | 63.9 | 1.8 | 3 | 2 |
| IIYGGSVTGATCK | TPI1 | 63.9 | 1.8 | 6 | 2 |
| YISPDQLADLYK | ENO1 | 63.9 | 1.8 | 2 | 2 |
| ECCEKPLLEK | ALB | 63.8 | 1.8 | 5 | 2;3 |
| SSLPEGIRPGTVLR | LGALS7 | 63.7 | 1.7 | 2 | 3 |
| GFSCGSAIVGGGKR | KRT4 | 63.6 | 1.7 | 5 | 2;3 |
| VGINYQPPTVVPGGDLAK | TUBA1C;TUBA4A;TUBA3E;TUBA3C;TUBA1A;TUBA1B;TUBA8 | 63.5 | 1.7 | 4 | 2 |
| QVESTQSMIR | ASCC3 | 63.5 | 1.7 | 2 | 2 |
| VATVSLPR | - | 63.5 | 1.7 | 20 | 2 |
| LDQGNLHTSVSSAQGQDAAQSEEK | CRNN | 63.4 | 1.7 | 4 | 3 |
| LKCDEWSVNSVGK | TF | 63.4 | 1.7 | 3 | 2;3 |
| ADDLGKGGNEESTK | SOD1 | 63.4 | 1.7 | 2 | 3 |
| YQPVSYKLCTR | AMY1A;AMY2B;AMY2A;AMY1B | 63.3 | 1.7 | 5 | 2;3 |
| FLIPNASQAESK | YWHAZ | 63.3 | 1.7 | 2 | 2 |
| ANRPFLVFIR | SERPINC1 | 63.3 | 1.7 | 2 | 3 |
| SLYNLRGNKSISMSVAGSR | KRT4 | 63.2 | 1.7 | 2 | 3 |
| QRPAEIKDYSPYFK | KRT14 | 63.2 | 1.7 | 16 | 2;3;4 |
| KAPDFVFYAPR | EZR;MSN;RDX | 63.2 | 1.7 | 2 | 3 |
| EYQELMNVK | KRT6A;KRT6C;KRT6B;KRT8 | 63.1 | 1.7 | 3 | 2 |
| FLEQQNKVLDTK | KRT5;KRT6C;KRT6B | 63.1 | 1.7 | 11 | 2;3 |
| DIQLARR | H3F3B;HIST2H3A;H3F3A;HIST1H3A;HIST3H3;H3F3C | 63.1 | 1.7 | 2 | 2 |
| LHQAMKGVGTR | ANXA1 | 63.1 | 1.7 | 6 | 2;3 |
| SMQDLVEDFKK | - | 63 | 1.6 | 7 | 2;3 |
| TEEREQIKLLNNK | KRT4 | 63 | 1.6 | 2 | 3 |
| TEITELRR | KRT13 | 63 | 1.6 | 4 | 2 |
| QFPFLASIQNQGR | AZU1 | 63 | 1.6 | 7 | 2;3 |
| CCPDTCGIKCLDPVDTPNPTR | SLPI | 62.9 | 1.6 | 4 | 3 |
| AADDTWEPFASGK | TTR | 62.8 | 1.6 | 3 | 2 |
| EIVNKHNELR | CRISP3;CRISP2 | 62.8 | 1.6 | 3 | 2;3 |
| AILVDLEPGTMDSVR | TUBB;TUBB3;TUBB2A;TUBB2B | 62.6 | 1.6 | 3 | 2 |
| SLPGQNEDLVLTGYQVDKNKDDELTGF | CSTA | 62.6 | 1.6 | 3 | 3 |
| RVVRDPQGIR | LYZ | 62.6 | 1.6 | 9 | 3 |
| AMVSEFLK | ANXA1 | 62.5 | 1.6 | 7 | 2 |
| KDLQNFLK | S100A9 | 62.5 | 1.6 | 8 | 2 |
| NWGEGWGFMPSDR | AMY1A;AMY2B | 62.5 | 1.6 | 3 | 2 |
| DASGATFTWTPSSGK | IGHA2 | 62.5 | 1.6 | 6 | 2 |
| FQLFGSPSGQKDLLFKDSAIGFSR | LTF | 62.4 | 1.5 | 7 | 3;4 |
| GFSCGSAIVGGGK | KRT4 | 62.3 | 1.5 | 6 | 2 |
| SQVVAGTNYFIK | CSTB | 62.3 | 1.5 | 9 | 2 |
| QEYDESGPSIVHR | ACTB;POTEE;POTEF;POTEI;POTEJ;POTEKP;ACTG1 | 62.2 | 1.5 | 2 | 3 |
